# A Case of Giant Cell Arteritis Presenting With a Hyperechoic Wall Thickening on Temporal Artery Ultrasonography

**DOI:** 10.7759/cureus.95118

**Published:** 2025-10-22

**Authors:** Katsuyuki Yoshida, Sotaro Jinnouchi, Takahiko Fukuchi

**Affiliations:** 1 Division of General Medicine, Department of Comprehensive Medicine 1, Saitama Medical Center, Jichi Medical University, Saitama, JPN; 2 Division of Cardiovascular Medicine, Department of Comprehensive Medicine 1, Saitama Medical Center, Jichi Medical University, Saitama, JPN

**Keywords:** atherosclerosis, giant cell arteritis, halo sign, polymyalgia rheumatica, temporal artery ultrasonography

## Abstract

Giant cell arteritis (GCA) is a type of systemic vasculitis in older adults that requires prompt diagnosis to prevent ischemic complications. Temporal artery (TA) ultrasonography is widely used as an initial diagnostic tool and typically demonstrates a hypoechoic halo sign. We report the case of a 91-year-old man with polymyalgia rheumatica who developed fever, malaise, proximal joint pain, and jaw claudication during steroid tapering. TA ultrasonography (linear 25 MHz, short-axis, non-compressible) revealed circumferential hyperechoic wall thickening rather than the typical halo sign, raising the suspicion of atherosclerotic changes. However, positron emission tomography-computed tomography demonstrated fluorodeoxyglucose uptake in the bilateral TAs and periarticular regions, and a TA biopsy confirmed GCA with lymphocytic infiltration and multinucleated giant cells. The patient was treated with high-dose prednisolone and tocilizumab and subsequently showed clinical improvement. This case highlights the atypical sonographic presentation of GCA mimicking degenerative arterial changes. Awareness of such atypical findings and the integration of clinical, imaging, and histopathological findings are crucial to avoid misdiagnosis and ensure timely management.

## Introduction

Giant cell arteritis (GCA) is a systemic vasculitis that affects adults over 50 years of age and primarily involves medium and large vessels, notably the temporal arteries (TAs) and axillary arteries [1]. Polymyalgia rheumatica (PMR) is a chronic inflammatory disease involving the periarticular structures of the proximal joints, almost exclusively in individuals older than 50 years [2]. Clinical overlap between the two conditions is common; manifestations of PMR are observed in 40-60% of patients with GCA, while 10-16% of patients with PMR eventually develop features of GCA [3]. Untreated GCA carries a significant risk of severe ischemic complications, including irreversible vision loss and stroke, making prompt diagnosis and treatment essential [4].

According to the European League Against Rheumatism (EULAR) recommendations, ultrasonography of the TAs and/or axillary arteries should be the first imaging modality in patients with suspected cranial GCA [5]. The halo sign is defined as a homogeneous, hypoechoic, noncompressible mural thickening caused by inflammatory edema of the intima-media. In contrast, hyperechoic intima-media changes are seen in atherosclerosis [4,5]. In GCA diagnosis, the halo sign demonstrates excellent specificity (96%; positive likelihood ratio 20.07) [6], while false-positive findings can occur due to various conditions, including atherosclerosis and other systemic diseases [7,8]. Although the halo sign sensitivity reaches 88% (negative likelihood ratio 0.13) [6], this is insufficient to confidently rule out GCA, and a negative finding should not be interpreted as sufficient to exclude GCA during differential diagnosis.

Furthermore, the main causes of false-negative results include partial steroid treatment, skip lesions, and technical factors [9]. Although the reliability of ultrasound findings is high among trained experts [5], its findings are considered highly operator-dependent [10]. Herein, we present an atypical case of GCA in which TA ultrasonography demonstrated hyperechoic wall thickening, a finding that challenged the classical sonographic presentation.

## Case presentation

A 91-year-old man presented with bilateral shoulder pain, fatigue, and elevated inflammatory markers. One year prior to his visit to our hospital, he had been diagnosed with PMR at a tertiary care center by the Department of General Medicine. Prednisolone (20 mg) treatment was initiated, resulting in a favorable initial response. However, during tapering, fatigue re-emerged at a prednisolone dose of 5 mg, and he developed fever and pain in both shoulders and hip joints at a dose of 2 mg. Approximately three weeks before the presentation to our hospital, he began experiencing jaw claudication, although he reported no visual or ocular symptoms. As symptom control was deemed inadequate by his previous physician, he was referred to our hospital for further evaluation.

His medical history included chronic atrial fibrillation, chronic heart failure, hypertension, Hashimoto thyroiditis, and osteoporosis. His current medications included prednisolone (1 mg/day), warfarin (2 mg/day), levothyroxine (50 µg/day), verapamil (80 mg/day), indapamide (1 mg/day), and alendronate (35 mg/week).

Upon physical examination at admission, his body temperature was 36.7 °C, blood pressure was 122/76 mmHg, heart rate was 94 beats per minute, respiratory rate was 22 breaths per minute, and oxygen saturation was 97% while breathing ambient air. The TAs were palpable and mildly indurated, without tenderness, and showed a loss of pulse. No tenderness was noted over the scalp or the temporomandibular joints. Tenderness was observed in the shoulder and gluteal regions. Laboratory tests revealed a white blood cell count of 8,740/µL (normal range 3,300-8,600/µL), a C-reactive protein level of 12.56 mg/dL (normal < 0.14 mg/dL), and an erythrocyte sedimentation rate of 112 mm/h (normal < 15 mm/h).

**Figure 1:**
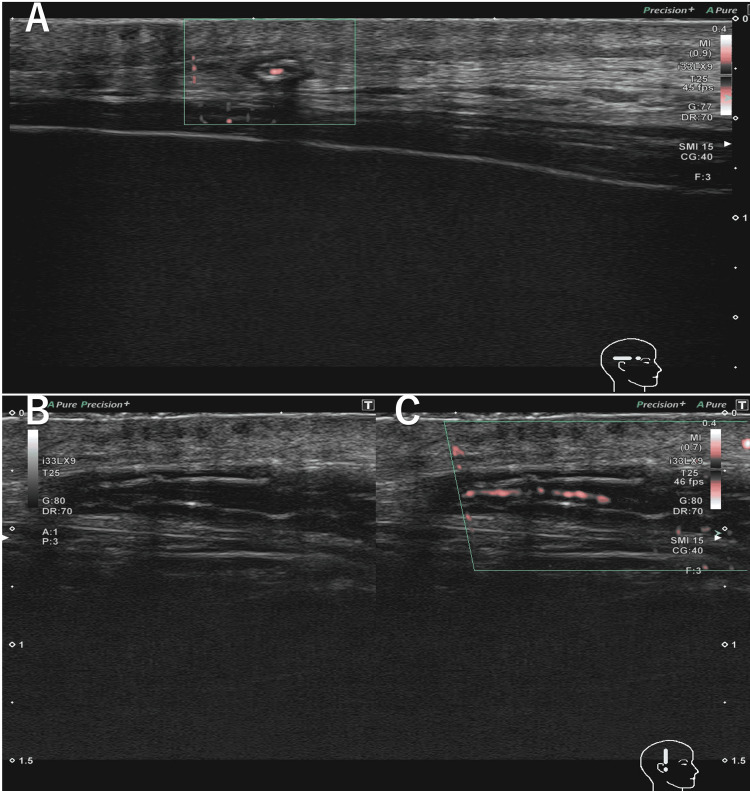
Temporal artery (TA) ultrasonography A. Color Doppler in the short-axis view (GG: 77 dB, CG: 40 dB, Frequencies: 15 MHz, PRF: 156 Hz) B. B-mode in the long-axis view (GG: 80 dB) C. Color Doppler in the long-axis view (GG: 80 dB, CG: 40 dB, Frequencies: 15 MHz, PRF: 156 Hz) TA ultrasonography shows right common temporal artery hyperechoic wall thickening circumferentially. CG: color gain, GG: grey gain, PRF: pulse repetition frequency

TA ultrasonography in the short-axis view showed bilateral circumferential hyperechoic wall thickening without compression (Canon Aplio i800 with i33LX9 linear transducer; 25 MHz) (Figure 1), and both the sonographer and radiologist reported that these findings were suggestive of atherosclerotic changes. Although the ultrasound report suggested atherosclerosis, the recent onset of jaw claudication and the loss of TA pulse suggest that GCA remained a possible diagnosis.

**Figure 2:**
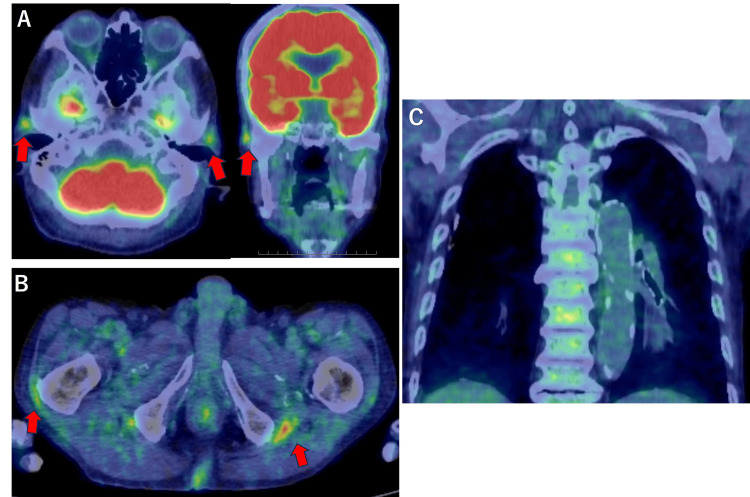
Positron emission tomography-computed tomography (PET-CT) A. FDG accumulation around the bilateral temporal arteries (Grade 2, red arrows) B. Hip joints and gluteal bursae (Grade 3, red arrows) C. Absence of FDG accumulation in the aorta (Grade 0) FDG uptake was qualitatively graded by visual comparison with the liver background FDG uptake (grading 0–3). FDG: fluorodeoxyglucose

Therefore, positron emission tomography-computed tomography (PET-CT) was performed, which revealed fluorodeoxyglucose (FDG) accumulation around the bilateral TAs, shoulder and hip joints, and gluteal bursae, consistent with PMR and GCA; however, no uptake was observed in the aorta (Figure 2).

**Figure 3:**
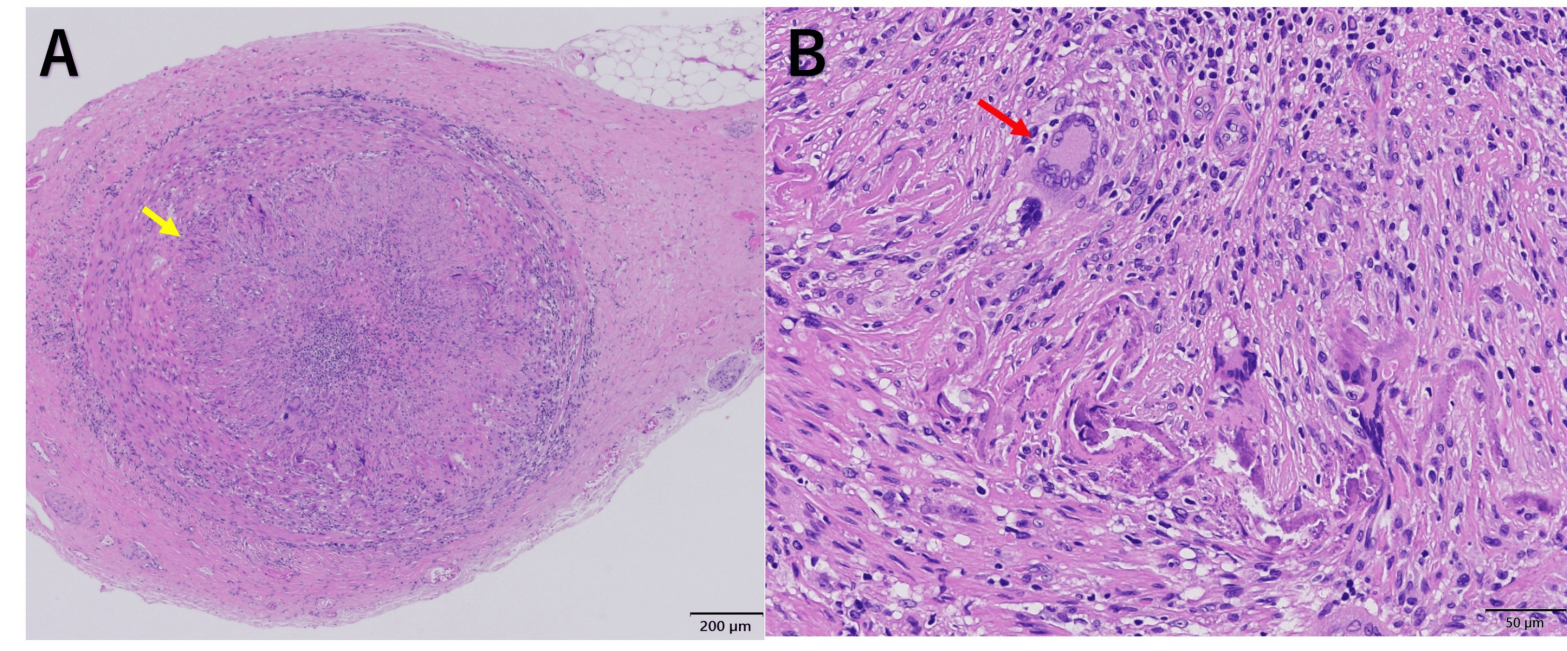
Right temporal artery biopsy specimen (pre-fixation length: 7.5 cm; 14 sections examined) A. H&E staining (×40) showing narrowing of the vascular lumen and disruption of the internal elastic lamina (yellow arrow) B. H&E staining (×200) demonstrating multinucleated giant cells in the tunica media (red arrow) H&E: hematoxylin and eosin

Based on the PET-CT findings, a biopsy of the right TA was performed two days after initiation of prednisolone (60 mg) therapy. Histopathological examination revealed lymphocytic infiltration with giant cells in the tunica media, along with a partial rupture extending from the media to the intima (Figure 3).

**Figure 4:**
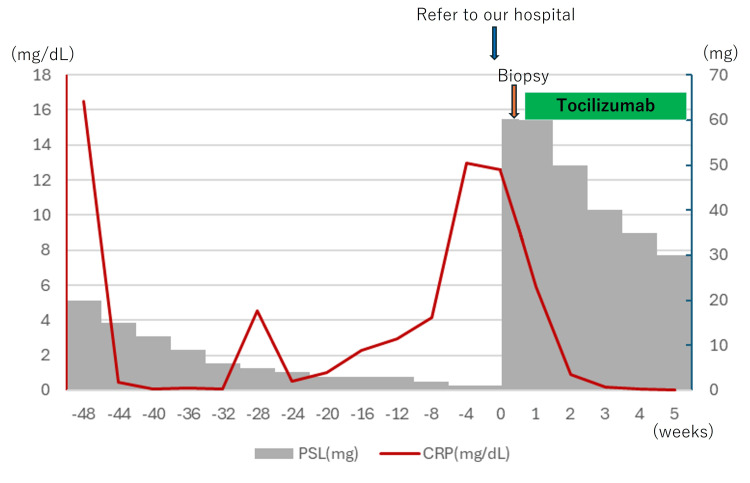
Clinical course of treatment CRP: C-reactive protein, PSL: prednisolone

Based on the imaging and pathology findings, a diagnosis of cranial GCA with coexisting PMR was established. Prednisolone (60 mg/day) treatment was initiated on day two of hospitalization, and tocilizumab was started on day five. Antiplatelet therapy was not added to reduce the risk of bleeding. Jaw claudication and pain in the shoulders and hips improved before discharge, and both C-reactive protein levels and the erythrocyte sedimentation rate had normalized by that time. The patient was discharged on day 21 without experiencing any major adverse events (Figure 4).

## Discussion

This case highlights an atypical presentation of GCA in which TA ultrasonography demonstrated hyperechoic wall thickening rather than the characteristic hypoechoic halo sign. Although the sonographer and radiologist who performed the TA ultrasonography reported these findings as atherosclerotic changes, the diagnosis of GCA was ultimately confirmed by TA biopsy. The decision to proceed with TA biopsy was guided by the patient's new-onset jaw claudication and increased FDG uptake in the TAs observed on PET-CT, which was inconsistent with PMR alone. As this is a single case, hyperechoic wall thickening should not be considered a diagnostic feature of GCA without histopathological or imaging corroboration. In this case, ultrasound evaluation of other large vessels, including the axillary arteries, was not performed, although these assessments may help differentially diagnose GCA.

The relationship between PMR and GCA has increasingly been conceptualized as a disease spectrum rather than as two separate entities. Cross-sectional and histopathological studies have shown that 15-20% of patients with PMR develop clinically overt GCA, and 16-21% of TA biopsy specimens from patients with PMR reveal histological features of vasculitis [11,12]. Furthermore, imaging studies suggest that 20-30% of patients with PMR exhibit subclinical large-vessel inflammation, potentially representing a preclinical stage of GCA [12]. Nonetheless, recent prospective cohort data indicate that the incidence of new-onset GCA during the first year after PMR diagnosis is relatively low (32 per 1000 person-years) [13]. In the present case, it remains uncertain whether GCA was present at the onset of PMR or emerged later. One possible explanation is that GCA developed during follow-up and was partially suppressed by corticosteroid therapy; however, as no follow-up ultrasonography or PET imaging was performed to support this inference, the explanation remains hypothetical.

The current European and U.S. guidelines differ in their recommended diagnostic approaches. The 2023 EULAR recommendations state that ultrasonography of the TAs and axillary arteries should be considered as the first-line diagnostic modality for suspected GCA [5]. The 2021 American College of Rheumatology guidelines continue to prioritize TA biopsy, reflecting concerns regarding operator expertise and test accessibility in routine practice [14]. TA biopsy has high specificity (95-99%) and variable sensitivity (38-79%) [15], depending on specimen length and prior corticosteroid exposure [16]. These differences highlight the need for individualized diagnostic strategies, depending on local expertise and resources.

Atherosclerosis is a well-recognized pitfall in the sonographic diagnosis of GCA. Both hypoechoic and hyperechoic intima-media thickening can mimic the halo sign, complicating its interpretation in elderly patients [8]. In contrast, false-negative halo signs may occur when the inflammatory process is segmental, when the involved arterial segment lies deeper, or when mural edema has already been reduced by corticosteroid treatment [5]. The halo sign itself reflects edema and inflammatory thickening within the intima and media; therefore, its absence does not reliably exclude active GCA [5]. The present case, in which hyperechoic wall thickening rather than the classical halo sign was observed, illustrates the diagnostic challenge of distinguishing GCA from degenerative arterial changes in partially treated or anatomically limited disease.

The natural course of vascular changes in GCA further complicates this interpretation. While the halo sign typically resolves with treatment, histopathological follow-up studies have demonstrated persistent inflammatory changes and increased medial fibrosis, even months after therapy [17]. In addition, ultrasonography studies of the axillary artery have shown that chronic lesions may appear as hyperechoic wall thickening resembling calcification that persists after clinical remission [18]. These observations may explain the atypical imaging findings in our patient, who had been receiving long-term corticosteroid therapy for PMR prior to the GCA diagnosis.

## Conclusions

This case of hyperechoic wall thickening on ultrasonography, while atypical, can indicate active GCA and should not be dismissed as mere atherosclerosis. This finding underscores the limitations of relying solely on TA ultrasonography in centers without specialized vascular sonography expertise. When atypical findings are encountered, careful integration of clinical features, disease course, and complementary diagnostic modalities (such as PET-CT or TA biopsy) is essential to avoid misdiagnosis and ensure timely management.
